# Artificial intelligence-assisted medical coding and DRG management: current applications, challenges, and future perspectives

**DOI:** 10.3389/fmed.2026.1913896

**Published:** 2026-08-04

**Authors:** Dandan Ji, Mingkui Huang, Liezhi Wang

**Affiliations:** 1 The First People's Hospital of Wenling, Taizhou, China; 2 Taizhou Central Hospital, Taizhou, China

**Keywords:** artificial intelligence, diagnosis-related groups, health informatics, large language models, medical coding, natural language processing

## Abstract

Artificial intelligence (AI) is reshaping the way medical information is processed and has shown considerable potential in medical record coding and diagnosis-related group (DRG) management. Traditional medical record management mainly relies on manual coding and rule-based matching, which is often limited by low efficiency, heavy workload, and insufficient consistency, making it difficult to meet the demands of large-scale healthcare data processing. In recent years, machine learning, deep learning, and natural language processing (NLP) have been increasingly applied to automated coding, clinical information extraction, and medical record quality control, and have gradually expanded to DRG grouping prediction, risk control, and hospital operation management. Large language models (LLMs) offer new opportunities for complex medical text understanding and candidate decision support; however, their application in medical record coding and DRG management remains at an early stage of validation and exploration. At present, this field still faces several challenges, including data heterogeneity, limited model interpretability, privacy and security concerns, and insufficient cross-institutional generalizability. Future efforts should focus on standardized validation, multimodal data integration, and human–AI collaboration mechanisms to promote the robust development of intelligent medical record management systems.

## 1 Introduction

In recent years, with the continued advancement of healthcare payment reforms based on diagnosis-related groups (DRGs), the quality of the front page of medical records and the accuracy of medical coding have become essential foundations for healthcare quality assessment, medical insurance reimbursement, and refined hospital management (1–3). Disease diagnosis codes and related clinical information not only directly determine DRG grouping results but are also widely used in healthcare quality monitoring and other management scenarios (4, 5). Therefore, improving the completeness, standardization, and accuracy of medical record data has become an important task in modern hospital management.

Traditional medical record coding mainly relies on professional coders to manually review electronic medical records, discharge summaries, and other relevant clinical documents before assigning diagnostic and procedural codes. With the widespread adoption of electronic medical record systems and the rapid growth of healthcare data, manual coding is increasingly challenged by heavy workload, low efficiency, insufficient coding consistency, and high training costs (6). In addition, coding proficiency varies across institutions, and undercoding, miscoding, and non-standardized coding remain common problems.

In recent years, artificial intelligence (AI) technologies have developed rapidly, particularly machine learning (ML), deep learning (DL), and natural language processing (NLP), which have been increasingly applied to medical information processing (7–9). By automatically analyzing electronic medical records and structured data, AI can assist in disease diagnosis information identification, coding recommendation, and medical record quality review, thereby providing a new technical pathway for intelligent medical record management (10). Meanwhile, large language models (LLMs), represented by GPT, DeepSeek, and other emerging systems, offer new possibilities for complex medical text understanding and candidate decision support. However, their reliability, interpretability, and real-world applicability still require further validation (11, 12).

Based on this context, this review summarizes the application progress of artificial intelligence in medical record coding and DRG management. We focus on its application scenarios in automated coding, clinical information extraction, medical record quality control, DRG grouping prediction, and hospital operation management. We also analyze the current technical challenges and practical issues in this field and discuss the application boundaries and validation requirements of LLMs in medical record management, with the aim of providing a reference for healthcare informatization and refined hospital management.

To summarize the research progress of artificial intelligence in medical record coding and DRG management, this review conducted a literature search using the Web of Science (WoS) and PubMed databases. The search covered publications from the past decade, from 2016 to 2026, with priority given to studies published in the most recent 5 years, from 2021 to 2026. Search terms were centered on topics including “artificial intelligence,” “machine learning,” “natural language processing,” “large language models,” “medical coding,” “ICD coding,” and “DRG management,” and were combined according to the search rules of each database. Literature screening was primarily based on thematic relevance and was manually evaluated by the researchers, with emphasis placed on studies closely related to medical coding, DRG prediction, and hospital information management. A narrative synthesis approach was adopted in this review, without involving systematic review procedures or quantitative analysis.

## 2 Applications of artificial intelligence in medical coding and DRG management

The application of artificial intelligence in medical record coding and DRG management does not follow a single technical pathway. Instead, it has formed multi-level application scenarios according to different management tasks. To avoid simply placing machine learning, deep learning, natural language processing, large language models, and business intelligence systems in parallel, this review summarizes the relevant studies from a task-oriented perspective, mainly including medical coding, clinical information extraction, medical record quality control, DRG grouping prediction, cost analysis, and hospital operation management.

To further present the data sources, model types, evaluation metrics, main findings, application stages, and limitations across different tasks, this review constructs a task-oriented evidence summary table (Table 1). This table provides a structured basis for the subsequent analysis of each application scenario.

**Table 1:** Task-oriented evidence summary of artificial intelligence applications in medical record coding and DRG management.

Task	Data sources	Model types	Evaluation metrics	Main findings	Application stage	Major limitations	References
ICD disease coding	MIMIC-III/hospital electronic medical records	NLP/transformer-based models/large language models	F1-score, accuracy	AI can help improve the consistency and automation of ICD coding, although results vary across studies	Retrospective studies/single-center validation	Limited cross-institutional generalizability; LLMs may produce hallucinations and remain dependent on coding standards	(24–26)
Surgical and procedural coding	Operative notes and clinical procedural texts	NLP/deep learning	Accuracy, recall	AI can help improve surgical coding efficiency and may reduce the risk of undercoding	Single-center retrospective studies	Surgical text expressions vary considerably, and standardization remains insufficient	(27–29)
Clinical information extraction	Discharge summaries/EMR systems	NER/BERT/transformer-based models	F1-score	AI can enable structured extraction of information on diseases, examinations, and medications	Retrospective data studies	High annotation costs; limited transferability across departments and languages	(30–32)
Medical record quality control/CDI	Medical record data/EHR data	Rule-based systems/ML/LLMs	Error detection rate, precision	AI can be used to identify documentation deficiencies, logical errors, and coding inconsistencies	Hospital pilots/retrospective analyses	Insufficient model interpretability; LLMs carry a risk of misjudgment	(33–35)
DRG grouping prediction	Medical insurance data/EHR data	ML, including XGBoost/DL/LLM-assisted methods	Accuracy, AUC	AI can be used to predict DRG grouping and support early resource allocation	Retrospective studies/partial pilot applications	Highly dependent on data quality; reduced transferability across systems	(36–38)
Risk stratification and anomaly detection	Historical hospitalization data/insurance claims data	Anomaly detection models/machine learning	AUC, sensitivity	AI can be used to identify high-cost and abnormal cases	Medical insurance review/research stage	Limited adaptability to emerging or atypical cases	(39–41)
Hospitalization cost prediction	Medical cost databases	Regression models/deep learning	MAE, MAPE	AI can be used to predict hospitalization costs and length of stay	Retrospective studies	Strong dependence on feature engineering; insufficient external validation	(42–44)
Hospital operation management: CMI and performance	DRG systems + KPI data	BI systems/AI analytics platforms	CMI, KPI indicators	AI/BI systems can support hospital operation analysis and resource allocation	Implemented applications/pilot applications	Inconsistent data standards; high costs of system integration	(45, 46)

The evaluation metrics listed in the table represent commonly used or literature-reported evaluation methods for the corresponding tasks. Because studies differ in data sources, coding systems, task definitions, model architectures, training strategies, and deployment environments, specific performance results should not be directly compared quantitatively across studies. As a narrative review, this article does not perform quantitative pooled analysis, but instead focuses on summarizing the types of evidence, evaluation metrics, application stages, and unresolved issues across different tasks.

### 2.1 Medical coding: ICD disease coding and surgical/procedural coding

Medical coding is a fundamental component of medical record management and DRG grouping, mainly including disease diagnosis coding and surgical/procedural coding. Disease coding usually relies on information from discharge diagnoses, progress notes, examination results, and the front page of medical records, whereas surgical and procedural coding depends more heavily on operative notes and perioperative documentation. Because clinical texts are often unstructured, highly variable in expression, and rich in specialized terminology, traditional manual coding is easily affected by coders’ experience, documentation quality, and differences in institutional coding practices, which may lead to undercoding, miscoding, or insufficient coding consistency.

In ICD disease coding, AI is mainly used to identify principal diagnoses, secondary diagnoses, complications, and comorbidities from electronic medical records through NLP, Transformer-based models, and related approaches, thereby generating candidate coding recommendations. Compared with early rule-based systems, semantic understanding-based models can better handle complex medical expressions. However, their performance still depends on the quality of training data, the consistency of coding standards, and local coding rules, and their cross-institutional generalizability remains limited.

In surgical and procedural coding, AI can identify procedure names, surgical sites, operative approaches, and relevant technical details from operative records and match them with standardized codes. Because surgical texts are highly dependent on specific procedural descriptions and clinical context, differences in physicians’ writing habits, terminology abbreviations, and missing procedural details may all affect model recognition performance. Therefore, at the current stage, the field of medical coding is more suited to a human–AI collaborative model characterized by “AI recommendation, coder review, and feedback-based correction,” rather than fully automated replacement of manual coding.

### 2.2 Clinical information extraction and structuring

Clinical information extraction is an important foundational task in intelligent medical record management. Its goal is to identify and extract key clinical information from unstructured electronic medical record texts for use in coding, quality control, and DRG analysis. A large amount of information in electronic medical records exists in natural language form, including diagnoses, procedures, examinations, medications, and treatment processes. If such information cannot be accurately structured, downstream tasks such as automated coding recommendation, medical record quality control, and DRG grouping prediction may all be affected.

Existing studies have commonly used named entity recognition, BERT, Transformer-based models, and related methods to identify entities such as diseases, surgical procedures, medications, laboratory tests, and imaging findings. Unlike tasks that directly generate coding results, information extraction places greater emphasis on the basic parsing of original medical record texts. Its outputs are usually structured fields or standardized clinical variables, which provide a data foundation for downstream tasks. However, medical record writing styles vary considerably across hospitals, departments, and physicians, and the construction of high-quality annotated datasets remains costly. Cross-institutional, cross-specialty, and cross-language transferability therefore remain important limitations in this field.

### 2.3 Medical record quality control and clinical documentation improvement

Medical record quality control is an important link connecting clinical documentation, medical coding, and DRG grouping results. Fields on the front page of medical records, such as the principal diagnosis, other diagnoses, surgical procedures, discharge disposition, and cost information, directly affect DRG grouping, medical insurance reimbursement, and hospital performance evaluation. Traditional quality control mainly relies on manual sampling and rule-based review. Although this approach has strong interpretability, it is limited by restricted coverage, delayed feedback, and a heavy manual workload.

AI can be used to identify missing fields on the front page of medical records, inconsistencies between diagnoses and procedures, unreasonable selection of principal diagnoses, and potential coding errors. Early systems mostly relied on rule engines and were suitable for detecting explicit logical errors. Machine learning and NLP models, by contrast, can integrate historical medical record data to support anomaly detection and risk alerts. In recent years, LLMs have enhanced the semantic understanding of clinical texts. However, the explanations generated by these models do not necessarily represent reliable clinical reasoning and may still be affected by prompt design, training data bias, and hallucinations. Therefore, at the current stage, AI is more appropriate as an auxiliary review tool for medical record quality control and still requires human verification and auditable mechanisms.

### 2.4 DRG grouping and risk analysis

DRG grouping prediction is an important application direction of AI in medical record management. Traditional DRG grouping usually depends on post-discharge diagnosis codes, surgical/procedural codes, and grouping rules, and therefore has a certain degree of delay. With the accumulation of electronic medical records, medical insurance reimbursement data, and hospital operation data, AI models can predict the DRG group into which a patient may be classified during hospitalization or before discharge, thereby providing references for clinical pathway management, resource allocation, and medical insurance risk control.

In model construction, DRG prediction usually uses patient demographics, principal diagnoses, comorbidities, complications, surgical procedures, laboratory and examination results, and historical cost data as input variables, with machine learning or deep learning models applied for classification prediction. In addition to grouping prediction, AI can also be used to identify high-outlier cases, low-outlier cases, cases with abnormal costs, and cases with suspected coding deviations, thereby assisting medical insurance review and internal hospital management. However, DRG-related models are highly sensitive to data quality, coding rules, and payment policies. Because DRG systems, medical insurance rules, and hospital workflows differ across regions, good performance in a single center does not necessarily mean that a model can be directly generalized. External validation, temporal validation, and local calibration are therefore still required.

### 2.5 Prediction of hospitalization costs and resource consumption

The prediction of hospitalization costs and resource consumption represents an important direction in which DRG management extends from grouping-based evaluation to refined hospital operation. Under the DRG payment model, case costs, length of stay, consumable use, and medical resource consumption not only influence medical insurance reimbursement but also reflect the implementation of clinical pathways and the level of cost control in hospitals. Therefore, using AI to predict hospitalization costs and resource consumption can help hospitals identify potential high-cost cases in advance and provide references for clinical department management and operational decision-making.

Existing studies have commonly used regression models, machine learning models, and deep learning methods, incorporating patient demographics, diagnosis codes, surgical procedures, comorbidities, laboratory test results, and historical cost data as input variables to predict hospitalization expenses, length of stay, or resource utilization levels. Compared with traditional statistical models, machine learning methods can capture nonlinear relationships among multiple factors. However, their application is still affected by charging standards, medical insurance policies, consumable use patterns, and differences in clinical pathways. In addition, cost prediction is highly dependent on feature engineering and data completeness, and most studies remain at the stage of retrospective modeling, with limited prospective validation and real-world intervention assessment.

### 2.6 Hospital operation management and decision support

As DRG management gradually evolves from a medical insurance payment tool into a tool for refined hospital operation management, the role of AI in performance evaluation, resource allocation, and management decision-making has attracted increasing attention. Compared with coding, quality control, and grouping prediction tasks, hospital operation management places greater emphasis on the integration of multi-source data, including front-page medical record data, DRG grouping results, the CMI, cost structure, departmental performance, bed utilization efficiency, and healthcare quality indicators.

In practical applications, business intelligence systems are mainly used for the integration, visualization, and dynamic monitoring of DRG-related indicators. When combined with AI methods such as machine learning, these systems can be further extended to trend prediction, anomaly detection, and risk early warning. However, AI applications at the hospital operation level still face challenges such as inconsistent data standards, complex system interfaces, and differences in indicator definitions. AI analytical platforms are therefore more suitable as auxiliary decision-support tools rather than replacements for direct managerial decision-making.

## 3 Application boundaries and validation requirements of large language models in medical record management

LLMs, represented by GPT, Claude, DeepSeek, and other systems, have attracted considerable attention in the field of medical text processing. Owing to their capabilities in complex language understanding, contextual integration, and text generation, LLMs may provide new auxiliary tools for medical record coding, medical record quality control, and DRG management (13–15). However, current evidence regarding their use in medical record management is still mainly derived from early experimental studies, task-specific validation, or exploratory applications, and there remains a considerable gap before mature clinical and managerial deployment can be achieved (16). Therefore, it is necessary to distinguish their general text-processing capabilities, early-stage auxiliary applications, and management decision-making functions that remain exploratory.

In the context of medical record coding, the potential value of LLMs is mainly reflected in medical text understanding, key information extraction, and candidate recommendation generation. They can assist in identifying principal diagnoses, secondary diagnoses, comorbidities, complications, and surgical/procedural information, and can generate candidate coding suggestions (17). However, candidate codes are not equivalent to final coding decisions. The selection of the principal diagnosis, determination of comorbidities and complications, matching of surgical/procedural codes, and application of coding rules still require consideration of the complete medical record context, coding standards, and professional review (18). Therefore, at the current stage, LLMs are more appropriately positioned as coding assistance tools rather than systems capable of independently completing medical coding.

In medical record quality control, LLMs can assist in identifying inconsistencies between clinical documentation and diagnostic codes, documentation deficiencies, and certain logical errors, and can generate revision suggestions or quality control alerts (15). However, their outputs may still be influenced by prompt design, training data bias, completeness of contextual input, and changes in model versions (19, 20). Particular attention should be paid to the fact that explanations generated by LLMs may appear superficially plausible, but they do not necessarily represent true, reliable, or traceable model reasoning. Therefore, LLM-generated explanations should not be directly equated with genuine interpretability, and their clinical validity and managerial usability still need to be verified through human review and auditable workflows.

In DRG management and hospital operation analysis, the application of LLMs is currently more exploratory. Their potential roles include assisting in the interpretation of medical record texts, summarizing case complexity, explaining DRG-related indicators, and identifying potential risks related to resource consumption (13, 17). However, DRG grouping prediction, cost prediction, abnormal case identification, and operational risk assessment still rely heavily on structured data, explicit grouping rules, medical insurance payment policies, and verifiable predictive models. Therefore, LLMs are more suitable as human–AI interaction interfaces, text explanation tools, or auxiliary analytical tools, and should be integrated with rule-based systems, machine learning models, and human review workflows.

In addition, the practical deployment of LLMs in medical record management still requires careful attention to hallucinations, prompt sensitivity, privacy protection, regulatory compliance, responsibility attribution, and human verification (21). Medical record data contain a large amount of sensitive health information, and model training, invocation, and deployment must meet data security and compliance requirements. When a model produces incorrect coding suggestions, erroneous quality control alerts, or inaccurate DRG-related analyses, how to define responsibility, trace the causes, and implement corrections remains a practical challenge (22, 23). Therefore, in the foreseeable future, the appropriate role of LLMs in medical record coding and DRG management should be that of a collaborative tool following the pathway of “assisted understanding, candidate recommendation, human review, and feedback-based optimization.”

## 4 Challenges and future perspectives

Although artificial intelligence has shown considerable potential in medical record coding and DRG management, the existing evidence remains markedly heterogeneous. Current studies vary substantially in data sources, coding systems, task definitions, model architectures, evaluation metrics, and deployment settings. Most studies are still limited to retrospective analyses, single-center validation, or specific task scenarios, with insufficient external validation, multicenter evaluation, and real-world deployment studies. This issue is particularly important in medical coding and DRG management, where institutions may differ in medical record writing habits, coding policies, medical insurance payment rules, language environments, specialty composition, and local management workflows, thereby limiting cross-institutional model generalization.

Therefore, future research should not focus solely on metrics such as accuracy, F1-score, AUC, or cost prediction error on internal test sets, but should also strengthen external validation, temporal validation, and cross-institutional validation. For medical coding tasks, differences among ICD-10, ICD-11, ICD-10-CM/PCS, and localized coding rules in different regions may all affect model transferability. For DRG management tasks, variations in DRG grouping rules, medical insurance payment policies, and hospital management workflows across countries and regions may also lead to performance degradation when models are applied across systems. In addition, policy adjustments, updates to coding guidelines, changes in disease spectra, and modifications to hospital workflows may all induce model drift. Therefore, in real-world applications, continuous performance monitoring, model recalibration, regular updating, and human review mechanisms are needed to ensure the stability and auditability of AI systems.

Medical coding and DRG management involve complex clinical judgment and managerial decision-making, and therefore require high levels of accuracy, interpretability, and auditability. At present, some AI models, particularly deep learning models and LLMs, still have certain “black-box” characteristics. When a model provides incorrect coding suggestions, abnormal quality control alerts, or DRG grouping predictions, how to trace the underlying causes and implement effective corrections remains an important issue. Meanwhile, medical record data contain a large amount of sensitive health information, and model training, data sharing, and system deployment must strictly meet requirements for privacy protection, regulatory compliance, and responsibility attribution.

Overall, the future development of AI in medical record coding and DRG management should not merely pursue improvements in model performance. Greater attention should be paid to real-world usability, interpretability, auditability, and the efficiency of human–AI collaboration. Future efforts should promote the standardized integration of electronic medical records, front-page medical record data, medical insurance payment data, and DRG indicators, while establishing secure, traceable, and updatable model management systems. In the foreseeable future, a collaborative model of “AI-assisted decision-making, professional review, and feedback-based correction” will remain the most realistic and feasible application pathway in medical record coding and DRG management.

## 5 Conclusion

With the continued advancement of DRG/DIP payment reform and healthcare informatization, medical record coding and DRG management have become increasingly important in healthcare quality evaluation, medical insurance reimbursement, and refined hospital operation. AI has gradually been applied to automated coding, clinical information extraction, medical record quality control, DRG grouping prediction, risk early warning, and operation management, providing new technical pathways for improving the efficiency of medical record data processing and supporting managerial decision-making.

However, the current application of AI in medical record coding and DRG management still faces challenges related to data quality, standardization, model interpretability, privacy and security, cross-institutional generalizability, and insufficient real-world validation. In particular, although LLMs offer new possibilities for medical text understanding, candidate coding recommendations, and quality control alerts, their reliability, auditability, and practical deployment value still require further validation. In the future, AI should be more robustly integrated into medical record management and hospital operation decision-making on the basis of standardized data governance, strengthened model validation, and improved human–AI collaboration mechanisms.
